# Functional analyses of male-specific early-phosphorylated proteins reveal an intrinsically disordered protein required for microgamete release in *Plasmodium berghei*

**DOI:** 10.3389/fcimb.2026.1900304

**Published:** 2026-08-05

**Authors:** Mark Tetteh-Tsifoanya, Takashi Sekine, Hidetaka Kosako, Tomoko Ishino, Naoaki Shinzawa

**Affiliations:** 1 Department of Parasitology and Tropical Medicine, Graduate School of Medical and Dental Sciences, Institute of Science Tokyo, Tokyo, Japan; 2 Division of Cell Signaling, Institute for Advanced Medical Sciences, Tokushima University, Tokushima, Japan

**Keywords:** fertilization, intrinsically disordered region, male gametogenesis, oocyst, phosphorylation, *Plasmodium berghei*

## Abstract

In the life cycle of the malaria parasite, *Plasmodium*, gametogenesis occurs upon the ingestion by mosquitoes of intraerythrocytic gametocytes circulating in the host bloodstream and subsequent fertilization in the mosquito midgut. Environmental changes, such as a drop in temperature and pH, and the influence of the mosquito midgut factor xanthurenic acid, induce male gametocytes to undergo multiple processes within 15 min; including emergence from within erythrocytes, three rounds of DNA replication, and the formation of flagellar gametes. A signaling pathway initiated by calcium-dependent protein kinase 4 in part facilitates these complex processes, but the detailed downstream mechanisms remain to be fully elucidated. Here, we investigated the functions of early-phosphorylated proteins upon the activation of male gametogenesis, to characterize the unique signaling cascade which facilitates *Plasmodium* sexual stage progression. Specifically, we focused on three proteins that are enriched in intrinsically disordered regions (IDRs) and are highly conserved across *Plasmodium* spp.; herein designated as male-specific early-phosphorylated IDR-rich proteins (MPDs). Gene disruption analyses via genome editing in the rodent malaria parasite *Plasmodium berghei* revealed that MPD2 plays an important role in microgamete release and subsequently affects oocyst formation in mosquito midguts. The observed reduction in microgamete release in ΔMPD2 was rescued by the introduction of a centromere plasmid containing an expression cassette for MPD2 fused with an AGIA-tag. Using these complemented parasites, we detected MPD2 expression in both gametocytes and gametes. These functional characterizations provide comprehensive insights into the unique mechanisms of rapid male gametogenesis triggered by environmental changes. This unique phosphorylation cascade may represent a promising target for developing novel transmission-blocking interventions.

## 1 Introduction

Malaria remains one of the most threatening infectious diseases worldwide, and causes over 200 million cases and more than 600,000 deaths annually (World Health Organization, 2025). The etiological agents, *Plasmodium* parasites, are transmitted to vertebrate hosts through the bite of infected *Anopheles* mosquitoes. During the asexual blood stage, a small percentage of intraerythrocytic parasites commit to developing into the transmissible sexual stage, and ultimately result in mature male and female gametocytes. Following ingestion from peripheral blood during a mosquito blood meal, they are rapidly activated to undergo transformation into functional gametes, termed gametogenesis, which is essential for subsequent fertilization and mosquito-stage development (Sinden, 1983). This activation is triggered by environmental cues within the mosquito midgut, including a drop in temperature, a rise in pH, and exposure to the insect-derived metabolite xanthurenic acid (Billker et al., 1998; Garcia et al., 1998; Jiang et al., 2020). Elucidating the underlying molecular mechanisms of gametogenesis is crucial for designing logical transmission-blocking strategies.

During gametogenesis, male and female gametocytes egress from within the parasite parasitophorous vacuole and the host erythrocyte membrane. The female gametocyte then matures into a single, spherical female gamete. In contrast, the male gametocyte undergoes three rapid rounds of mitosis and axoneme assembly, culminating in the release of eight flagellated male gametes, a process known as exflagellation. This differentiation process is completed within 10 to 15 minutes, and is tightly regulated by a rapid, dynamic cascade of intracellular signaling pathways, particularly post-translational protein phosphorylation. Calcium-dependent protein kinase 4 (CDPK4) functions as a master regulator (Billker et al., 2004; Kumar et al., 2021), dominantly during male gametogenesis. Downstream of CDPK4 activation, distinct phosphorylation signals mediated by various protein kinases and phosphatases drive multiple cellular events in a parallel and coordinated manner; such as host cell egress, DNA replication, and flagellar assembly (Tewari et al., 2010; Guttery et al., 2014; Brochet and Billker, 2016).

To analyze these instantaneous signaling dynamics, a recent study utilized high-resolution mass spectrometry to establish a sub-minute phosphoproteomic dataset during *Plasmodium berghei* gametogenesis (Invergo et al., 2017). This study showed that numerous proteins are phosphorylated or dephosphorylated within 20 seconds of activation, regulating processes such as DNA replication, mitosis, and flagellar assembly. Although this dataset provided an overview of the early phosphoregulation network, the majority of these rapidly phosphorylated proteins are functionally uncharacterized. Therefore, it remains unclear which specific molecules regulate the initiation and progression of early male gametogenesis.

In this study, we screened the sub-minute phosphoproteomic dataset to identify key regulators governing the initiation of male gametogenesis. Through this screening, we focused on three uncharacterized proteins that exhibit robust and immediate phosphorylation within 60 seconds post-activation, and are preferentially expressed in male gametocytes. Structural modeling using AlphaFold3 revealed that all three candidates are highly enriched in intrinsically disordered regions (IDRs). Using CRISPR/Cas9-mediated gene disruption, we demonstrated that the loss of one of these IDR-rich proteins significantly impaired the progression of exflagellation after male gametocyte activation and subsequent fertilization.

## 2 Materials and methods

### 2.1 Experimental animals, parasites, and mosquitoes

The experimental protocols involving animal subjects were reviewed and approved by the Animal Experiment Committee of the Institute of Science, Tokyo. ICR mice were maintained under controlled environmental conditions with a 12-hour light/dark cycle and ambient temperature. All transgenic parasites were derived from the *Plasmodium berghei* pbcas9 strain, which constitutively expresses Cas9 nuclease (Shinzawa et al., 2020) and were propagated in female ICR mice, aged 4 to 6 weeks. *Anopheles stephensi* SDA500 mosquitoes maintained in our laboratory (Oundavong et al., 2026) were used for blood-feeding experiments. Larval and adult mosquitoes were maintained at 25 °C and 70% humidity under a 12-h light/dark cycle. Adults were fed a 5% (w/v) sucrose solution.

### 2.2 Construction of centromere plasmid vector

A male-specific mScarlet-expressing centromere plasmid was constructed using a backbone plasmid containing the *P. berghei* centromere sequence (Yuda et al., 2010). A 1.5-kb promoter region of the male-specific dynein heavy chain (PBANKA_0416100) was amplified with primers dyn-5UTR-F and dyn-5UTR-R. Using a synthesized DNA fragment (GenScript) as a template, a DNA fragment encoding mScarlet-I was amplified with primers mSca-F and mSca-R. These two fragments were then co-inserted into KpnI/BglII-digested pCen-GFP using the In-Fusion Snap Assembly Master Mix (Takara Bio, Japan). For the complementation of *mpd2* (PBANKA_0508600), the *mpd2* promoter region (~1.0 kb) and open reading frame (ORF) were amplified using primers mpd2-5UTR-F and mpd2-ORF-R, while the *mpd2* 3’UTR was amplified using primers mpd2-3UTR-F and mpd2-3UTR-R. These fragments were amplified to share overlapping homologous sequences encoding an AGIA-tag (Morita et al., 2021), and were subsequently co-inserted into KpnI/SalI-digested pCen-GFP.

### 2.3 Construction of sgRNA-expressing plasmid and preparation of donor template DNA

The construction of single guide RNA (sgRNA)-expressing plasmids and the preparation of donor template DNA were performed as reported (Shinzawa et al., 2020), with slight modifications. Briefly, target sequences for the guide RNAs were designed using the CHOPCHOP program (https://chopchop.cbu.uib.no) to minimize potential off-target effects. The designed oligonucleotide DNAs were annealed and cloned into the BsmBI-digested psgRNA1 vector. For the preparation of donor template DNA for homology-directed repair, a two-step overlap PCR approach was employed. In the first round of PCR, homologous sequences containing a premature stop codon were introduced to the ends of the fragments. These fragments were then utilized as templates for the second round of PCR to generate the final donor DNA. All oligonucleotide DNAs used in this study are listed in **Supplementary Table 1**.

### 2.4 Generation of transgenic parasites

Transfection of *P. berghei* was performed as described with some modifications (Shinzawa et al., 2020; Oundavong et al., 2026). Briefly, 10 µg of a sgRNA plasmid and 10 µg of linear donor DNA were introduced into purified schizonts of pbcas9 parasites using the FI-115 program on a 4D-Nucleofector device (Lonza, Switzerland). After electroporation, the parasites were immediately injected intravenously into ICR mice, and pyrimethamine selection of transgenic parasites was initiated 30 hours after transfection by administering the drug via drinking water (70 µg/mL) for 5 days. Parasites successfully obtained after drug withdrawal were evaluated for correct editing using genotyping analysis. For the introduction of the centromere plasmid, 5 µg of the plasmid was used for the transfection of pbcas9 and ΔMPD2. After electroporation, parasites harboring the plasmid were similarly selected using pyrimethamine in the drinking water.

### 2.5 Genotyping PCR

Genotyping PCR was performed using mouse blood containing parasites that emerged after drug withdrawal. Tail blood diluted 5-fold with distilled water was used as the PCR template. Primers were designed to bind to the respective target gene regions, with one primer located outside the donor DNA homology region and the other near the insertion site. Twenty cycles of PCR amplification was conducted using Takara Ex Premier DNA Polymerase. The PCR products were resolved by electrophoresis on a 1.5% agarose/TAE gel at 100 V for 30 min, stained with GelGreen^®^ (Fujifilm Wako, Japan), and visualized. The amplified fragments were then subjected to direct Sanger sequencing to confirm the successful insertion of the stop codon.

### 2.6 Re-analysis of RNA-sequencing data for sex-specific transcriptomes

To compare mRNA expression levels between male and female parasites, a published sex-specific transcriptome dataset (Yeoh et al., 2017) was re-analyzed. Fastq files from RNA-sequencing of male, female, and asexual parasites (BioProject accession number: PRJNA374918) were mapped to the *Plasmodium berghei* ANKA reference genome from PlasmoDB-68 using HISAT2 (version 2.2.1), with the maximum intron length parameter set to 2,000. To accurately quantify gene expression, a cDNA-only GFF file was generated from the *P. berghei* ANKA GTF file obtained from PlasmoDB-68. Read counts for each gene were subsequently quantified from the mapped data using featureCounts (version 2.0.1) from the Subread package. From the resulting read counts, Transcripts Per Kilobase Million (TPM) values were calculated to evaluate gene expression levels.

### 2.7 AlphaFold-based structural analysis

Amino acid sequences were retrieved from PlasmoDB (https://plasmodb.org), and protein structures were predicted using AlphaFold3 (https://alphafoldserver.com/). Single-structure visualization and structural superimpositions were performed using PyMOL. The intrinsically disordered region (IDR) percentage was defined as the proportion of amino acids with a predicted local distance difference test (pLDDT) score of <50. Predicted Alignment Error (PAE) plots were obtained directly from the AlphaFold Server outputs.

### 2.8 Gametocyte purification and male gametocyte sorting

Female ICR mice were treated with phenylhydrazine and intravenously injected with 2×10^5^ plasmid-harboring parasites, as described (Beetsma et al., 1998). To impose plasmid retention the injected mice were maintained on drinking water with pyrimethamine (70 µg/mL). To eliminate asexual stage parasites, sulfadiazine (15 μg/mL) treatments were administered via drinking water for 2 days starting on day 3 post-infection. On day 5 post-infection, gametocyte-enriched blood was collected from mice via cardiac puncture and suspended in RPMI 1640 medium supplemented with 25% FCS. For gametocyte purification, a density-gradient working solution was prepared by mixing Optiprep (Serumwerk, Germany) and 1.25x RPMI-1640 medium at a 1:4 ratio. Prewarmed working solution was gently introduced beneath the prewarmed cell suspension and centrifuged at 300 × g for 25 min at 37 °C under conditions that prevent gametocyte activation. The interface containing the purified gametocytes was collected and washed with prewarmed RPMI 1640 medium containing 25% FCS. The collected gametocytes were then centrifuged at 1000 × g for 10 min at 37 °C, and the supernatant was discarded. The cell pellet was resuspended with prewarmed RPMI 1640 and subjected to cell sorting based on the male-specific mScarlet fluorescence signal using an SH800S cell sorter (Sony, Tokyo, Japan) configured with a “Normal” sorting purity mode and a sample pressure setting of 10. Data were analyzed and visualized using FlowJo software (BD Biosciences). The sorted male gametocytes were then pelleted by centrifugation and one half of the cell pellet was immediately snap-frozen in liquid nitrogen and stored as the pre-activation (0 min) sample. The remaining half of the pellet was resuspended in exflagellation medium (RPMI 1640 medium containing 100 µM xanthurenic acid, 50 mg/L hypoxanthine, 25 mM HEPES, 24 mM NaHCO_3_, and 20% FCS; adjusted to pH 7.4) and incubated at 20 °C for 5 min to induce activation. Following the 5-min incubation, the sample was immediately chilled on ice to halt the activation process, pelleted by centrifugation, and snap-frozen. These frozen cell pellets were subsequently used for the downstream phosphoproteomic analysis.

### 2.9 Phosphoproteomics

Roughly 3 × 10^6^ male parasites were used for phosphoproteomic analysis as reported (Haku et al., 2026). Briefly, the samples were lysed in 6 M guanidine buffer containing 100 mM HEPES-NaOH (pH 7.5), 10 mM TCEP, and 40 mM chloroacetamide. The lysates were heated at 95 °C for 5 min, sonicated, and clarified by centrifugation. Proteins were precipitated using a methanol/chloroform-based procedure and resuspended in 0.1% RapiGest SF (Waters) in 50 mM triethylammonium bicarbonate. After sonication and heat treatment, proteins were digested overnight at 37 °C with trypsin/Lys-C mix (Promega). The resulting peptides were acidified with trifluoroacetic acid, and phosphopeptides were enriched using a High-Select Fe-NTA Phosphopeptide Enrichment Kit (Thermo Fisher Scientific). Enriched phosphopeptides were desalted using GL-Tip SDB (GL Sciences), eluted with 50% acetonitrile/0.1% trifluoroacetic acid, concentrated by vacuum centrifugation, and reconstituted in 3% acetonitrile/0.1% trifluoroacetic acid. LC-MS/MS analysis was performed on a nanoElute 2 coupled to a timsTOF HT mass spectrometer (Bruker) using a data-dependent acquisition-parallel accumulation serial fragmentation (DDA-PASEF) method. The acquired raw data were processed using FragPipe against the *Plasmodium berghei* and *Mus musculus* protein database. Carbamidomethylation of cysteine residues was set as a fixed modification, whereas oxidation of methionine and phosphorylation of serine, threonine, and tyrosine residues were set as variable modifications.

### 2.10 Male and female differentiation

Female ICR mice were treated intraperitoneally (i.p.) with 200 µL of phenylhydrazine (6 mg/mL) for three days prior to infection and were then injected intravenously with 2×10^5^ of pbcas9 or mutant (ΔMPD1, ΔMPD2, and ΔMPD3) parasites. On day 5 of infection, parasitemias were counted by microscopy using glass slides of Giemsa-stained thin smears of tail blood. Male and female gametocytes were identified based on published criteria (Kaneko et al., 2023). On the Giemsa-stained smears, mature female gametocytes were identified as mononuclear cells with bluish-purple cytoplasm and a compact nucleus, while mature male gametocytes exhibited faintly stained cytoplasm and a diffused nucleus.

### 2.11 Exflagellation assay

Exflagellation assays were performed as reported with some modifications (Shinzawa et al., 2020). Female ICR mice treated with phenylhydrazine were intravenously injected with 2×10^5^ of pbcas9 or mutant (ΔMPD1, ΔMPD2, and ΔMPD3) parasites. On days 4 to 6 post-infection 3 μL of infected mouse blood was mixed separately with 57 μL of exflagellation medium to activate male gametocytes. Samples were mixed gently and loaded onto a hemocytometer and incubated for 8 mins at 20 °C. After incubation, samples were analyzed by light microscopy to count the number of exflagellation centers per 1×10^5^ RBCs (Blagborough and Sinden, 2009).

### 2.12 Oocyst formation

To initiate infection, 4-week-old female ICR mice were intravenously injected with 2×10^4^ pbcas9 or ΔMPD2 parasites. Five days later, mice with fully mature gametocytes (defined as having >30 exflagellation centers per 1×10^5^ RBCs) were used for blood-feeding of adult female 5-to-7-day-old *Anopheles stephensi* mosquitoes. Fully engorged mosquitoes were selected and maintained at 20 °C until dissection. On day 13 post-feeding, midguts were isolated from mosquitoes, and stained with 0.5% mercurochrome solution for 10 mins and washed in 50% glycerol in PBS for 10 mins. The stained midguts were then mounted on a glass slide with 50% glycerol in PBS, covered with a coverslip, and the number of midgut oocysts were counted using a BX53 upright microscope (Olympus, Japan).

### 2.13 Western blotting

Purified gametocytes were used for Western blotting analysis. For the non-activated control, gametocyte pellets were immediately resuspended with RBC lysis buffer (154.4 mM NH_4_Cl, 10 mM KHCO_3,_ and 0.1 mM EDTA-2Na) supplemented with a protease inhibitor cocktail (P8340, Sigma-Aldrich) and incubated for 5 min on ice. After washing twice with 1× D-PBS, the samples were solubilized in 1.5× SDS sample buffer (Nacalai Tesque, Kyoto, Japan). For gametocyte activation, gametocyte pellets were resuspended in 600 µL of exflagellation medium, divided into three tubes, and incubated at 20 °C for 5, 10, and 30 min. Immediately after incubation, all samples were transferred onto ice and centrifuged at 1500×*g* for 4 min at 4 °C, followed by removal of the supernatant. The resulting pellets were subjected to RBC lysis and subsequent solubilization in 1.5× SDS sample buffer. To shear genomic DNA samples were sonicated using a Bioruptor II (Cosmo Bio, Tokyo, Japan), then 5% β-mercaptoethanol was added and the samples were incubated at 4 °C overnight. Electrophoresis was performed using a specified quantity of parasites on 5–20% polyacrylamide gradient gels (ATTO, Tokyo, Japan), followed by transfer to polyvinylidene difluoride (PVDF) membranes using a semi-dry system. Membranes were blocked overnight with 4% skim milk and subsequently incubated for 90 minutes at room temperature with primary antibodies at the following dilutions: mouse anti-AGIA antibodies (1 µg/mL) (Oundavong et al., 2026), rabbit anti-PyMiGS antiserum (1:5,000) (Tachibana et al., 2018), and rabbit anti-PbHSP70 antiserum (1:400,000) (Ishino et al., 2019). Horseradish peroxidase (HRP)-conjugated goat anti-rabbit IgG (H+L) (11-035-144, 1:10,000 dilution, Jackson ImmunoResearch Laboratories, USA) and HRP-conjugated goat anti-mouse IgG (H+L) (115-035-146, 1:10,000 dilution, Jackson ImmunoResearch Laboratories) were applied as secondary antibodies for 60 minutes under identical conditions. Chemiluminescence detection was conducted using Immobilon Western Chemiluminescent HRP Substrate (Millipore, USA), with signals visualized and captured using an iBright CL1500 Imaging System (Thermo Fisher Scientific).

### 2.14 Phylogenetic analysis

Amino acid sequences were retrieved from PlasmoDB. Multiple sequence alignments were generated using MAFFT with the --localpair --maxiterate 1000 parameters. Based on the resulting alignments, a phylogenetic tree was constructed by the Maximum Likelihood method using MEGA12. Node support was evaluated by bootstrap analysis with 1,000 replications.

### 2.15 Statistical analysis

Statistical analyses for the phenotypic evaluation of the ΔMPD2 parasite line were conducted using GraphPad Prism software (version 9.0.0). Statistical significance was determined using a one-way ANOVA followed by Dunnett’s *post-hoc* test for multiple comparisons. For the comparison of oocyst numbers, the Mann-Whitney U non-parametric test was performed.

### 2.16 Ethics statement

All animal experiments were approved by the Institutional Animal Care and Use Committee of the Institute of Science Tokyo (Approval No. A2025-149A, A2026-088A) and were conducted in accordance with relevant guidelines and regulations consistent with the ARRIVE guidelines (https://arriveguidelines.org). Mice were euthanized by gradual-fill carbon dioxide (CO_2_) inhalation using procedures consistent with the AVMA Guidelines for the Euthanasia of Animals (2020 Edition) (https://www.avma.org/resources-tools/avma-policies/avma-guidelines-euthanasia-animals).

## 3 Results

### 3.1 Identification of male-specific early-phosphorylated intrinsic disordered proteins

**Figure 1:**
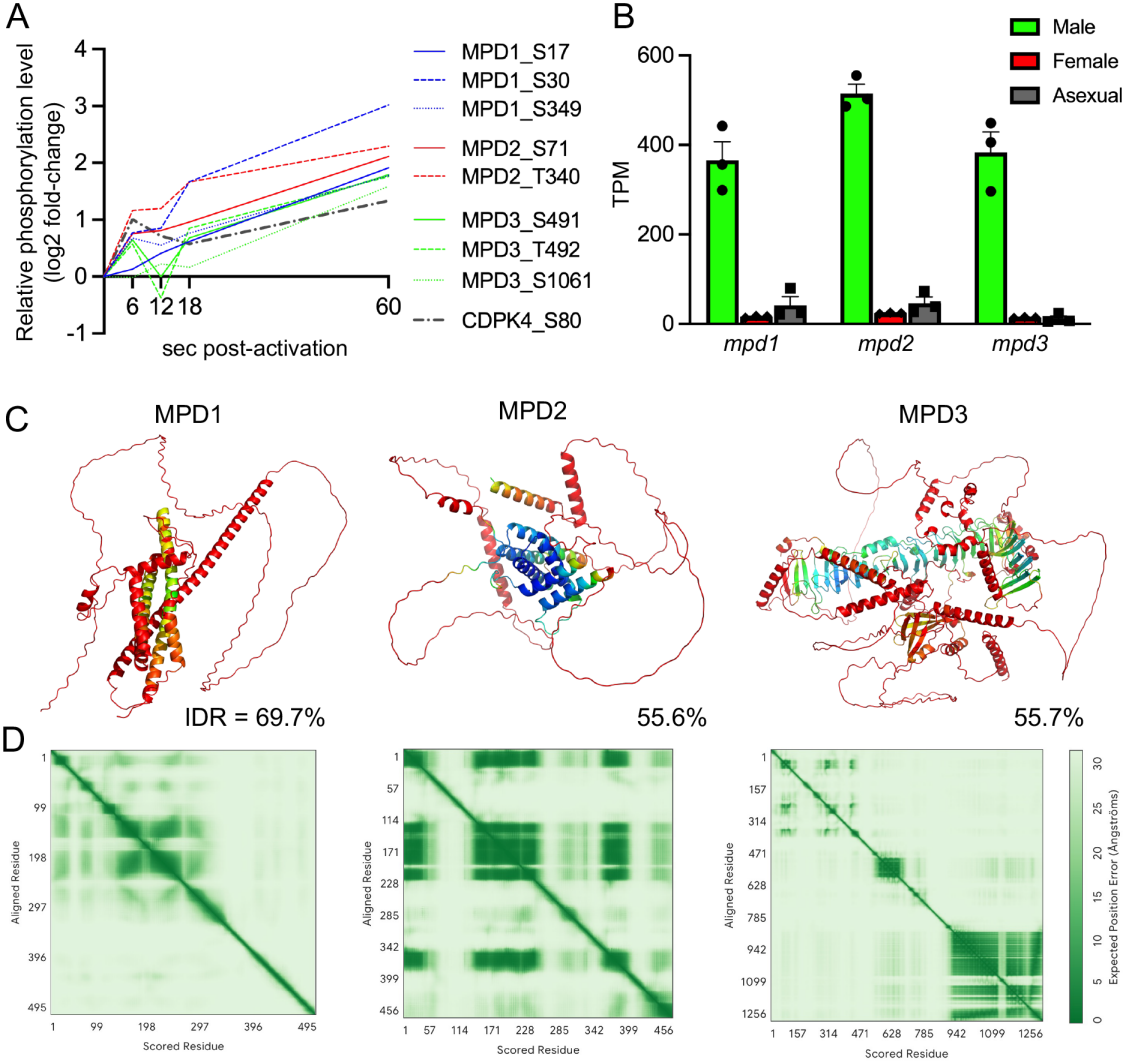
Selection of male-specific early-phosphorylated IDR-rich proteins (MPDs). **(A)** Phosphorylation kinetics of MPD1–3 following gametocyte activation. Relative phosphorylation levels of MPD1 (PBANKA_0302800), MPD2 (PBANKA_0508600), and MPD3 (PBANKA_1405000) are plotted at 0, 6, 12, 18, and 60 s post-activation. A typical responsive protein (CDPK4; PBANKA_0615200) is shown as a control. Data were retrieved and plotted from the published phosphoproteomic dataset (**Supplementary Table 3** of Invergo et al., 2017). **(B)** mRNA expression profile in male and female gametocytes, and erythrocytic stages. Relative expression levels of MPD1, MPD2, and MPD3 were re-analyzed using reported RNA-sequencing data (Yeoh et al., 2017). TPM (Transcripts Per Million) were recalculated by the HISAT2-featureCounts pipeline. Average TPMs are shown as bars. **(C)** Predicted structural models of MPD1, MPD2, and MPD3 generated by AlphaFold3. The models are colored with a rainbow gradient in PyMOL based on the pLDDT confidence score, spanning from high confidence (pLDDT > 90) to very low confidence (pLDDT < 50), with the latter corresponding to intrinsically disordered regions (IDRs). All three proteins are predominantly composed of IDRs with low pLDDT scores. **(D)** Predicted Aligned Error (PAE) plots for the MPD1, MPD2, and MPD3 models. The large lightly shaded areas across all three plots indicate high structural flexibility, supporting their classification as IDPs.

To identify key regulators of early gametogenesis, we used a published sub-minute phosphoproteomic dataset to screen for proteins phosphorylated immediately upon activation of gametocytes (Invergo et al., 2017). Numerous proteins exhibited rapid phosphorylation following a reduction in temperature and the addition of xanthurenic acid; and we focused on three candidates, PBANKA_0302800, PBANKA_0508600, and PBANKA_1405000, which are conserved across *Plasmodium* species but remain uncharacterized. Their phosphorylation was confirmed to be robust within 60 seconds of activation (**Figure 1A**). A significantly higher mRNA expression in male gametocytes (**Figure 1B**) was determined by analysis of published RNA-sequencing data for the sex-specific transcriptome (Yeoh et al., 2017). Domain architecture analysis showed that one of the candidates, PBANKA_1405000, possesses MORN (Membrane Occupation and Recognition Nexus) repeats, whereas no distinct functional domains were identified in the other two proteins. To investigate the structural properties of these three candidates, we performed AlphaFold3-based modeling. The predicted models for all three proteins exhibited prominently low pLDDT confidence scores (represented in orange), revealing that they are predominantly composed of intrinsically disordered regions (IDRs) (**Figure 1C**). This highly flexible nature was further supported by their respective Predicted Aligned Error (PAE) plots (**Figure 1D**). Based on these collective features, we designated these proteins as male-specific early-phosphorylated IDR-rich proteins 1, 2, and 3 (MPD1-3), respectively.

### 3.2 Isolation of high-purity male gametocytes for quantitative phosphoproteomic validation

**Figure 2:**
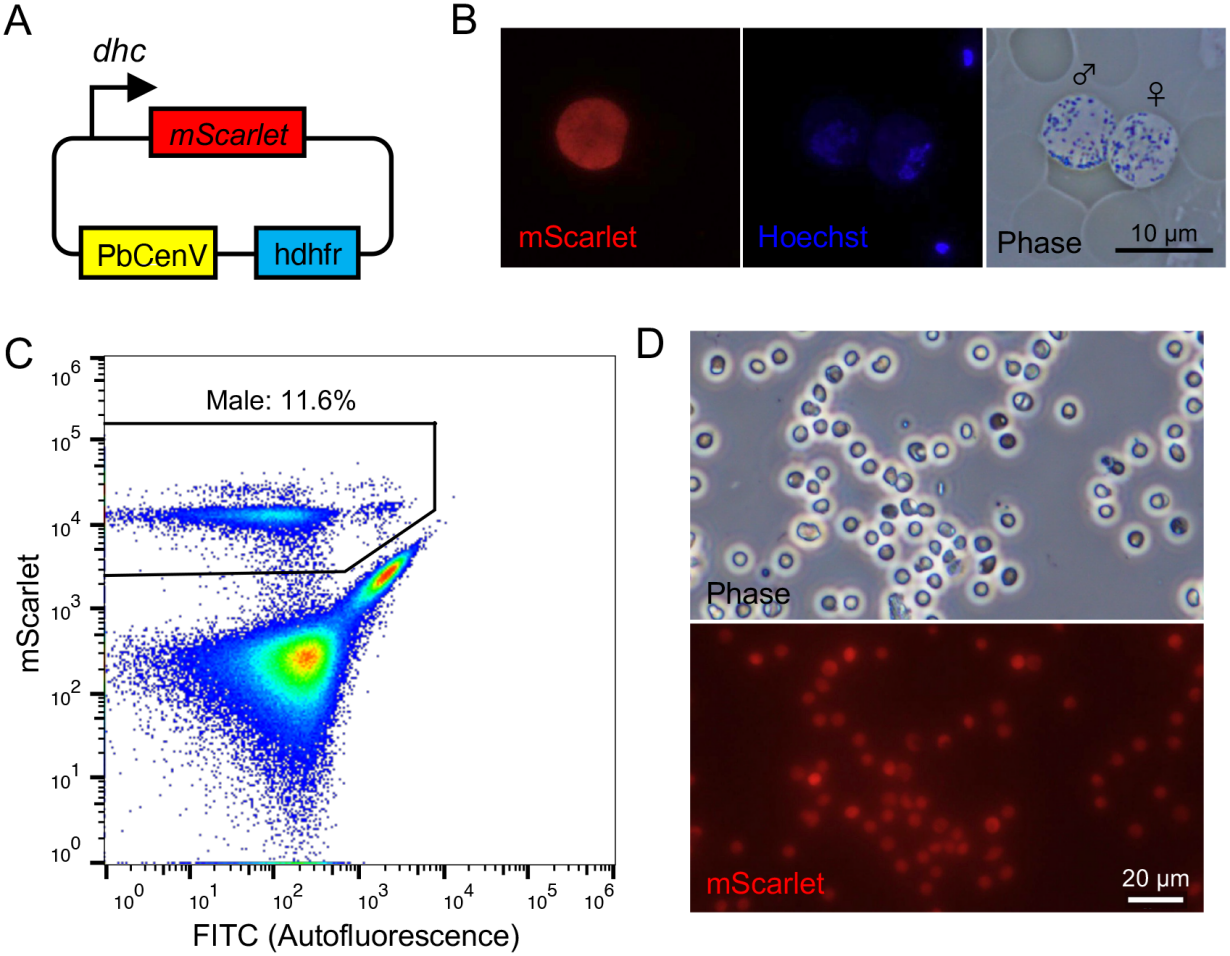
Isolation of male gametocytes utilizing male-specific mScarlet-expressing parasites. **(A)** Schematic representation of the transfection vector design for male-specific mScarlet expression. The promoter of the gene encoding male-specific dynein heavy chain (*dhc*; PBANKA _0416100) was used for mScarlet transcription. **(B)** Representative fluorescence microscopic images of the transgenic parasites expressing male-specific mScarlet in mouse blood smears. Male gamatocytes exhibited robust mScarlet signals, whereas no fluorescence was detected in female gametocytes. **(C)** FACS gating strategy for sorting male gametocytes using an SH800S cell sorter based on mScarlet fluorescence. The plot displays 200,000 cell events and was generated using FlowJo software. **(D)** Microscopic confirmation of the sorted pellet, demonstrating a high proportion of mScarlet-positive cells.

To characterize the phosphorylation kinetics of male-specific proteins MPD1, MPD2, and MPD3 during *Plasmodium* activation, a high-purity isolation workflow was established for male gametocytes. A transgenic parasite line was generated which harbors the mScarlet expressing vector driven by the male-specific dynein heavy chain promoter, and its male-specific fluorescence was confirmed (**Figures 2A, B**). Utilizing this fluorescent marker, male gametocytes were successfully separated and collected via flow cytometry-based cell sorting (**Figure 2C**). Microscopic observation confirmed that the majority of sorted cells were mScarlet-positive, thus verifying sample purity (**Figure 2D**). Using these highly enriched male gametocyte samples, high-resolution MS/MS was performed to track the quantitative changes in target phosphopeptides at 0 and 5 min post-activation. As summarized in **Table 1**, targeted phosphopeptides derived from MPD1, MPD2, and MPD3 were successfully identified with high confidence; demonstrating clear up-regulation or maintenance of phosphorylation levels at 5 min post-activation. Several critical phosphorylation sites on MPD2 (e.g., S71 and T340) and MPD3 (e.g., S491, T492, and S1061) displayed rapid, multifold increases in intensity or were exclusively detected after the 5-min activation trigger, highlighting their potential involvement in male gametogenesis.

**Table 1:** High-resolution MS/MS identification and quantitative kinetics of MPD phosphopeptides detected at 5 min post-activation.

Protein ID/target site^a^	Identified phosphopeptide sequence^b^	Intensity (0 min)	Intensity (5 min)	Fold change (5m/0m)^c^
PBANKA_0302800 (MPD1)				
– S30	YSIN**pS**FNYK	112,723	108,160	0.96
– S349	NMIPSSTD**pS**LVGGNLNQFEK	1,025,651	1,063,383	1.04
– S349	N(ox)MIPSSTD**pS**LVGGNLNQFEK	267,682	340,648	1.27
PBANKA_0508600 (MPD2)				
– S71	KIF**pS**LGLLSNEKDK	0	114,096	N.A.
– S71	IF**pS**LGLLSNE	0	48,749	N.A.
– S71	IF**pS**LGLLSNEKDK	117,810	857,025	7.27
– T340	NIADNNLSSSD**pT**INIK	402,519	1,019,940	2.53
– T340	NIADNNLSSSD**pT**INIKK	481,284	548,773	1.14
PBANKA_1405000 (MPD3)				
– S491	MRpT**pS**TFFSGKPSEDPTK	211,050	316,155	1.5
– S491	(ox)MRpT**pS**TFFSGKPSEDPTK	0	274,288	N.A.
– T492	MRpTS**pT**FFSGKPSEDPTK	0	260,211	N.A.
– S1061	LNDQHEFNL**pS**LSDTK	714,682	3,745,349	5.24

^a^ Target phosphorylation sites focused on in this validation study, selected based on the external baseline phosphoproteomics data (Invergo et al., 2017).

^b^ Lowercase “p” indicates identified phosphorylation sites; the phosphorylated residues are shown in bold. “(ox)” indicates methionine oxidation.

^c^ N.A., Not Applicable due to zero intensity at the 0 min baseline.

### 3.3 Generation of gene-disrupted mutants of MPDs by CRISPR/Cas9-mediated genome editing

**Figure 3:**
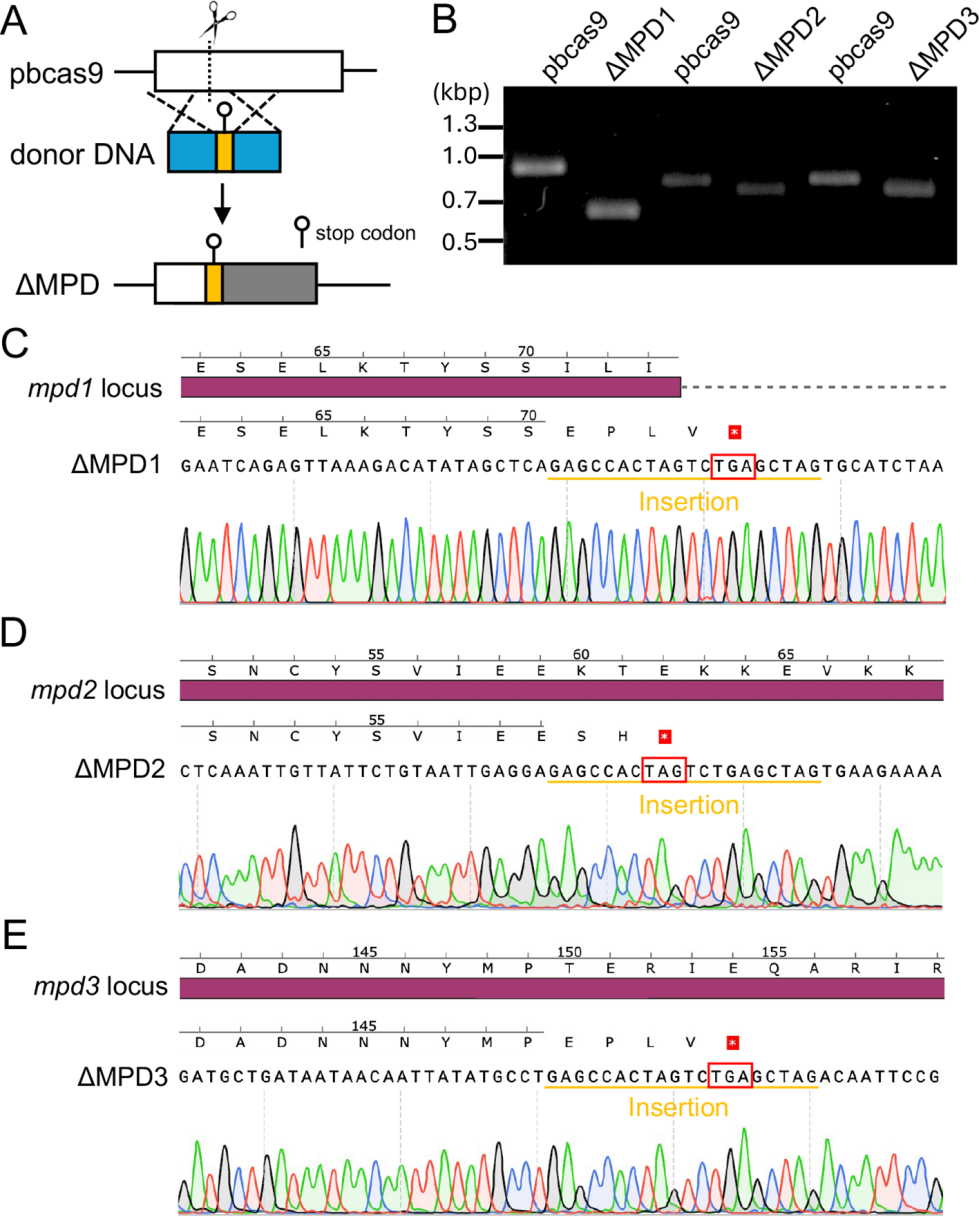
Generation of gene disruption mutants of MPDs by CRISPR/Cas9-mediated genome editing. **(A)** Schematic diagrams of genome editing. pbcas9 parasites were co-transfected with PCR-synthesized donor DNA fragments and sgRNA-expressing plasmids. **(B)** Genotyping PCR of the parasites that emerged following drug withdrawal. DNA fragments were amplified using the primer set designed to bind outside of the homologous regions. Wild-type signals were below the detection threshold. **(C–E)** Sanger sequencing of the transfected parasite populations. In-frame stop codons were correctly introduced into the MPD1 **(C)**, MPD2 **(D)**, and MPD3 **(E)** loci.

To elucidate the roles of MPDs in male gametogenesis, we performed targeted gene disruption using the CRISPR/Cas9 system to introduce internal deletions of 80 to 300 bp coupled with a premature stop codon (**Figure 3A**). For each target gene, Cas9-expressing *P. berghei* parasites (Shinzawa et al., 2020) were transfected with a linear donor DNA and a sgRNA-expressing plasmid to achieve genome editing. Transfectants were selected via five days of pyrimethamine treatment. PCR performed on the parasite population that emerged after drug withdrawal confirmed the absence of a diagnostic wildtype PCR product, indicating that the resulting parasite population consisted of mutated parasites (**Figure 3B**). The intended genomic modifications were confirmed by Sanger sequencing of the target locus in each mutant line (**Figures 3C**–**E**). Consequently, these bulk transfectant populations were utilized as gene-disrupted mutants in all subsequent analyses, and were designated as parasite lines ΔMPD1, ΔMPD2, and ΔMPD3.

### 3.4 Impaired normal male gametogenesis in ΔMPD2

**Figure 4:**
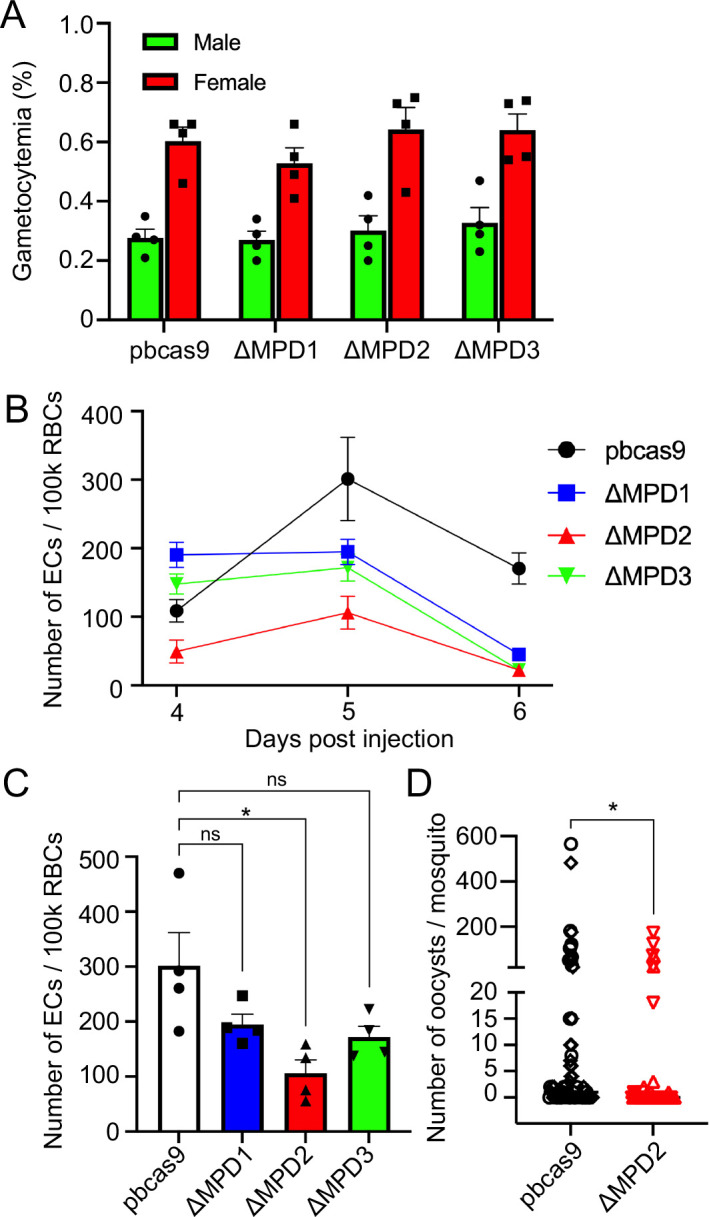
Phenotypic analyses of the MPD-disrupted mutants ΔMPD1, ΔMPD2, and ΔMPD3. **(A)** Male and female gametocyte emergence. Sex-specific identification was conducted by Giemsa staining. The mutants exhibited comparable gametocyte commitment to the parental line. Error bar indicates SEM (n=3). **(B)** Number of exflagellation centers (ECs) quantified after *in vitro* activation at indicated days post-injection. Error bar indicates SEM (n=4). RBC, red blood cell. **(C)** Exflagellation center numbers on day 5 post-infection, extracted from **(B)**. The ΔMPD2 mutants showed a significantly reduced number of exflagellation centers. *p < 0.01, ns: not significant using Dunnett’s multiple comparison test following one-way ANOVA. **(D)** The number of oocysts at 12 days after direct feeding on infected mice. ΔMPD2 mutants showed a significantly reduced level of oocyst formation. *p < 0.0001 using the Mann-Whitney test. Data are pooled from two independent feeding experiments for each strain, with different symbols representing individual experiments.

Phenotypic analyses were performed to characterize the physiology of the parasite lines ΔMPD1, ΔMPD2, and ΔMPD3. For all lines male and female gametocyte formation proceeded normally, as assessed by Giemsa-stained blood smears (**Figure 4A**). The abilities to initiate gametogenesis were determined by assaying exflagellation center formation following activation. While ΔMPD1 and ΔMPD3 showed no significant defects, the ΔMPD2 mutants exhibited a significantly reduced rate of exflagellation (**Figure 4B**), with a prominent defect observed at day 5 post-infection (**Figure 4C**). Consistent with this defect in male gametocyte activation, the number of oocysts per mosquito midgut was also significantly diminished in the ΔMPD2 line compared to the parental control (**Figure 4D**). These results indicate that MPD2 is required for the efficient progression of male gametogenesis and subsequent fertilization.

### 3.5 MPD2 is required for efficient male gametogenesis

**Figure 5:**
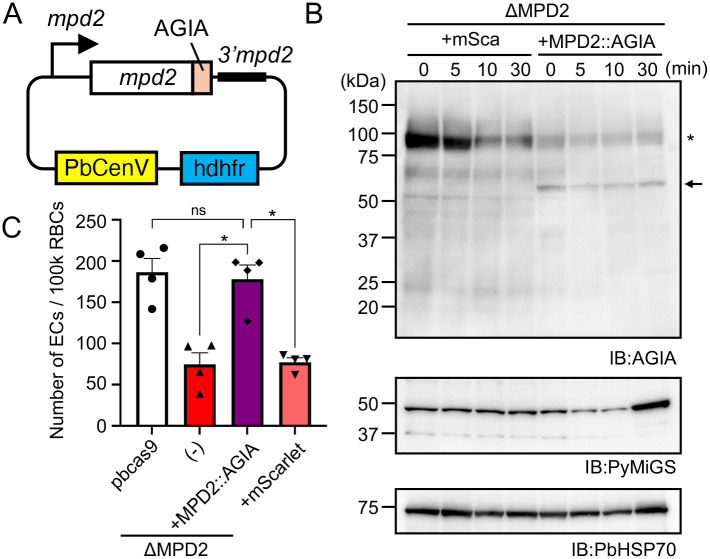
MPD2 is responsible for normal male gametogenesis. **(A)** Map of the complementation vector to express MPD2. The vector consists of 5’UTR, ORF, and 3’UTR of MPD2, AGIA-tag, centromere (CenV), and a drug-selectable cassette (hDHFR). **(B)** Western blotting analysis of the MPD2-complemented parasites. Parasites transfected with an mScarlet-expression plasmid served as a negative control. PyMiGS and PbHSP70 were used as the internal loading control for gametocytes and total parasites, respectively. Samples were loaded equivalent to 1 × 10^7^ parasites for AGIA, and 2 × 10^6^ parasites for PyMiGS and PbHSP70. An arrow indicates MPD2::AGIA. An asterisk indicates non-specific bands. **(C)** Number of exflagellation centers quantified after *in vitro* activation at day 5 post-injection of the MPD2-complemented parasites. The complementation restored the defect of normal gametogenesis in ΔMPD2. Error bar indicates SEM (n=4). *p < 0.001, ns: not significant using Dunnett’s multiple comparison test following one-way ANOVA.

To confirm that the observed phenotypes were specifically caused by the loss of MPD2, a complementation experiment was performed using a centromere plasmid. Since centromere plasmids are maintained at a stable copy number in *Plasmodium* cells (Iwanaga et al., 2010), they minimize cell-to-cell variation and are ideal for rescue experiments. A complementation vector was constructed which contains an MPD2 promoter, ORF, and 3’ UTR (**Figure 5A**). The MPD2 ORF was fused with a C-terminal AGIA tag, which has been shown to be a highly efficient epitope tag in *Plasmodium* (Morita et al., 2021). Using the AGIA tag, the expression of the MPD2 protein in intact and activated male gametocytes was determined by Western blotting (**Figure 5B**), which confirmed that the MPD2 protein was successfully expressed from the complementation vector in the parasites. The episomal expression of MPD2 restored the exflagellation rate to levels comparable to the parental pbcas9 line, demonstrating that the loss of MPD2 was indeed responsible for the impaired exflagellation of ΔMPD2 (**Figure 5C**). These results suggest that MPD2 is phosphorylated upon activation and plays a crucial role in the progression of male gametogenesis.

### 3.6 Phylogenetic and structural characterization of MPD2

**Figure 6:**
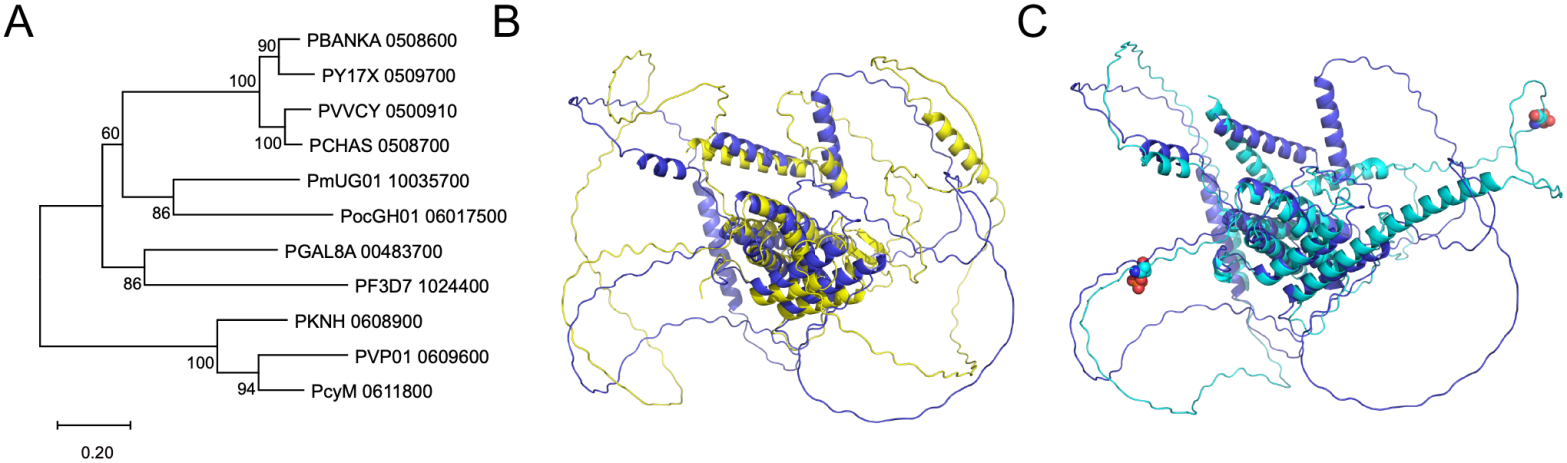
Phylogenetic and structural analyses of MPD2. **(A)** Phylogenetic tree of the amino acid sequences of PbMPD2 (PBANKA_0508600) and its orthologs in the genus *Plasmodium*; specifically, *P. yoelii*, *P. chabaudi*, *P. vinckei*, *P. knowlesi*, *P. cynomolgi*, *P. falciparum*, *P. vivax*, *P. malariae*, *P. ovale*, and *P. gallinaceum*. The alignment was generated using MAFFT, and the tree was constructed by MEGA12 using the Maximum-likelihood method. Numbers at the nodes represent bootstrap percentages based on 1000 replications. **(B)** Structural superimposition of PbMPD2 (blue; shown in **Figure 1C**) and PfMPD2 (yellow) performed using PyMOL. This comparison supports the idea that both proteins share an extensively disordered profile despite their sequence divergence. **(C)** Predicted structural model of phosphorylated PbMPD2 (ppPbMPD2;cyan; pS71 and pT340) superimposed with the unphosphorylated form (blue). The introduced phosphate groups are highlighted as spheres. This analysis shows no dramatic conformational changes within the IDR regions.

An investigation of the evolutionary conservation and structural properties of MPD2 was conducted and phylogenetic analysis revealed that MPD2 is specifically conserved within the genus *Plasmodium*, with no clear orthologs identified in other apicomplexan parasites or higher eukaryotes (**Figure 6A**). While the local primary sequence identity reached approximately 51% between orthologs within *P. berghei* (PbMPD2) and *P. falciparum* (PfMPD2), with a query coverage of 21%, the overall length and basic amino acid composition remain relatively conserved. This genus-specific conservation suggests that MPD2 may have evolved to fulfill a specialized role unique to the *Plasmodium* life cycle, such as the rapid transition required for fertilization within the mosquito midgut.

To investigate the structural basis of its function, we performed a superimposition of the predicted PbMPD2 structure with the *P. falciparum* ortholog, PfMPD2; which supported the idea that both proteins share an extensively disordered profile despite their sequence divergence (**Figure 6B**), with an IDR content of 55.6% for PbMPD2 and 60.4% for PfMPD2 (**Figure 6B**). Modeling of the phosphorylated PbMPD2 (ppPbMPD2; pS71 and pT340) and subsequent superimposition with the unphosphorylated form revealed that while the overall backbone topology exhibited no dramatic conformational changes, the IDR content decreased to 42.9% (**Figure 6C**); indicating a localized, phosphorylation-induced structural stabilization. Together, these structural insights indicate that the highly flexible framework, rather than a strictly rigid conformation, is an evolutionarily conserved and functionally essential characteristic of the MPD2 protein among *Plasmodium* species.

## 4 Discussion

In this study we investigated the functional impact of early phosphorylated proteins on male gametogenesis in *Plasmodium* parasites. We established a new high-resolution phosphoproteomic dataset encompassing this early phase, thereby complementing and functionally validating reported rapid phosphoproteomic dynamics (Invergo et al., 2017). Three proteins were selected for characterization based on their robust phosphorylation and structurally predicted intrinsically disordered regions (IDRs). One of them, MPD2, was demonstrated to play an important role in efficient microgamete release. Given that hundreds of proteins undergo rapid phosphoregulation and multiple cellular events proceed in parallel during male gametogenesis (Alonso-Morales et al., 2016; Balestra et al., 2020), MPD2 might regulate a specific subset of these signaling pathways. Further analyses focusing on the identification of interacting partners or downstream substrates of MPD2 are required to elucidate the precise downstream pathways that culminate in exflagellation.

Structural modeling using AlphaFold3 revealed that MPD1, MPD2 and MPD3 contain high proportions of IDRs. These identified IDRs in *Plasmodium* proteins are often correlated with low-complexity regions characterized by significant amino acid biases; indeed, this feature is prominent across the *Plasmodium* proteome, as documented since early genome annotations (Aravind et al., 2003; Feng et al., 2006). Due to their inherent conformational flexibility, IDR-containing proteins are well-documented to function as signaling hubs (Wright and Dyson, 2014). The requirement of MPD2 for efficient microgamete release underscores the functional importance of IDRs in the rapid signaling networks governing *Plasmodium* male gametogenesis. In particular, phosphorylation within IDRs often act as instantaneous switches to modulate interactions with binding partners through changes in protein charge configurations (Iakoucheva et al., 2004; Wright and Dyson, 2014; Holehouse and Kragelund, 2023). The rapid and widespread phosphorylation of these IDR-rich proteins within 20 to 60 seconds post-activation represents a highly efficient system to drive and facilitate the signaling network required to complete DNA replication and flagellar assembly simultaneously within a highly compressed 10-to-15-minute window. Although MPD1 and MPD3 did not exhibit prominent phenotypes upon disruption, they may nevertheless operate as minor yet interconnected components within this extensive, IDR-mediated signaling meshwork. In particular, because MORN repeats are domains implicated in membrane binding or cytoskeletal organization (Takeshima et al., 2000), MPD3 may help interact with or stabilize specific proteins while the male gametocyte undergoes structural remodeling. The lack of a phenotype for these two proteins may be due to gene redundancy, meaning that other similar proteins can compensate for the loss of MPD1 or MPD3. A similar phenomenon has been reported for PfMORN1, another MORN-repeat protein in *Plasmodium falciparum*, which is dispensable for asexual blood-stage replication likely due to functional redundancy within the basal complex molecular machinery (Moran and Dvorin, 2021).

Our structural comparison of the phosphorylated and unphosphorylated forms of MPD2 indicated that while the protein undergoes a localized structural stabilization upon phosphorylation, it largely retains its flexible, disordered framework. This localized transition suggests that complete folding or further rigidification of MPD2 may depend on integrated regulatory inputs, requiring additional environmental or intracellular cues such as changes in temperature, ambient pH, or local calcium concentrations (Alberti et al., 2019). The subtle yet distinct structural rearrangement observed upon phosphorylation carries critical functional implications. Phosphorylation-induced alterations in the charge configuration of MPD2 IDRs may influence its subcellular localization or partitioning behavior independently of conformational change (Bah and Forman-Kay, 2016). For instance, such electrostatic shifts could modulate the propensity of the protein for phase separation, potentially governing its transition between condensed and dispersed states within the cell (Mitrea and Kriwacki, 2016). As computational structural biology tools, such as AlphaFold, evolve to incorporate these diverse environmental parameters, the precise prediction of conditional structural dynamics within IDRs, including charge-driven changes in phase separation behavior, will likely become more efficient and achievable (Ruff and Pappu, 2021; Brotzakis et al., 2025; Abramson et al., 2024).

Numerous highly conserved eukaryotic protein kinases are known to orchestrate male gametogenesis in both *P. berghei* and *P. falciparum*; such as calcium-dependent kinase, cyclin-dependent kinase, and mitogen-activating kinase (Billker et al., 2004; Tewari et al., 2005; Balestra et al., 2020; Bansal et al., 2018; Hitz et al., 2020; Kumar et al., 2022). Previous sub-minute phosphoproteomic datasets of CDPK4-knockout parasites did not highlight MPD2 as a prominent downstream target (Invergo et al., 2017; Fang et al., 2017). However, it is critical to note that these comparative analyses were strictly limited to an ultra-early timepoint (18 seconds post-activation) where the activation-dependent phosphorylation of MPD2 was not yet robustly detectable even in wild-type controls. Given that CDPK4 operates as a master genetic regulator indispensable for the simultaneous events of DNA replication, axoneme assembly, and egress (Billker et al., 2004; Fang et al., 2017), elucidating the precise hierarchical relationship between CDPK4 and MPD2 presents a compelling avenue for further investigation, particularly during the subsequent functional phase such as the 5-minute window validated in our study.

In contrast to the ubiquitous and conserved protein kinases that drive upstream signaling cascades, MPD2 is uniquely restricted to the genus *Plasmodium* and is absent in other apicomplexan parasites. Our comparative analysis demonstrates that MPD2 is phylogenetically well-conserved across the *Plasmodium* genus. This evolutionary conservation, along with the shared architecture of an IDR-rich framework and a rigid central core between PbMPD2 and PfMPD2, strongly implies that this molecule constitutes a core component of a fundamental signaling pathway essential throughout the genus; and may represent an evolutionary adaptation specific to the mosquito-borne lifecycle of *Plasmodium*. Elucidating such *Plasmodium*-specific molecular foundations will provide crucial insights for the rational design of future transmission-blocking strategies.

## Data Availability

The original contributions presented in the study are included in the article/**Supplementary Material**. Further inquiries can be directed to the corresponding author.
